# A multilevel conceptual framework for dementia: from life-course mechanisms to public health strategies

**DOI:** 10.1590/1414-431X2026e15447

**Published:** 2026-08-17

**Authors:** M.J.R. Aliberti, J.E. Paiva

**Affiliations:** 1 Instituto de Pesquisa, Hospital Sírio-Libanês, São Paulo, SP, Brasil; 2 Laboratório de Investigação Médica em Envelhecimento (LIM-66), Serviço de Geriatria, Hospital das Clínicas, Faculdade de Medicina, Universidade de São Paulo, São Paulo, SP, Brasil; 3 Johns Hopkins Bloomberg School of Public Health, Baltimore, Maryland, USA

**Keywords:** Dementia, Life-course, Conceptual framework, Cognitive reserve, Cerebrovascular health, Multilevel determinants

## Abstract

Dementia is a growing global health challenge driven by population aging and unequal exposure to biological, behavioral, social, and environmental determinants across the life course. Existing evidence demonstrates that these factors accumulate and interact from fetal development through old age, shaping cognitive reserve, brain resilience, and vulnerability to neurodegeneration. However, current models rarely integrate these multilevel influences in a structured manner. This study proposes a comprehensive multilevel conceptual framework for dementia that maps determinants across seven levels (biological, behavioral, psychological, individual, family, community, and national) and across all life stages, from prenatal development to late life. The framework is organized along a bi-axial structure that aligns levels of influence with temporal windows of exposure, allowing a clear visualization of how early-life and midlife factors contribute to dementia in older age. Two illustrative pathways are explored in depth. The first describes the development of cognitive reserve, highlighting the roles of maternal health, education, health literacy, social engagement, and lifelong learning in delaying symptom onset despite neuropathological burden. The second focuses on cerebrovascular integrity, demonstrating how prenatal vascular development, lifestyle behaviors, and cardiovascular risk profiles influence vascular cognitive impairment and interact with Alzheimer-related pathologies. The framework distinguishes modifiable population-level determinants, such as education quality and public health policies, from more fixed biological factors, thereby identifying critical intervention points. By integrating life-course epidemiology with multilevel determinants, this model provides a structured approach for understanding dementia etiology and supports the development of targeted prevention strategies across individual, community, and societal domains.

## Introduction

As populations age worldwide, dementia has become an enormous challenge, with studies estimating that 57 million people live with dementia globally (1). This number is expected to double every 20 years, reaching 152 million people by 2050, with 71% living in low- and middle-income countries (LMICs) (2). This sharp increase impacts individuals and societies economically, as the annual global cost of dementia is tremendously high, calculated at USD 1.3 trillion in 2019 (2). Dementia is characterized by a gradual decline in memory, language, and problem-solving abilities, which makes this condition a leading cause of years lived with disability (YLD), imposing a significant burden on families, caregivers, and healthcare systems (3).

Dementia results from the cumulative influence of genetic, biological, social, and environmental factors over the life course (4). While advanced age is a major risk factor, exposures at different life stages, such as education and social conditions, also play a critical role in shaping cognitive reserve and brain resilience, protecting against dementia in old age (5). A life-course perspective is essential for understanding how these early influences affect cognitive outcomes later in life (6). Moreover, these factors can be classified at different levels according to their proximity and influence on the outcome, ranging from biological and behavioral to community and national levels (7).

A conceptual framework for dementia must therefore be broad and integrative, encompassing the genetic, biological, behavioral, psychosocial, and environmental factors that contribute to risk (4,6). In this study, we propose a multilevel life-course model that incorporates established and emerging risk factors across the life span, illustrating how their cumulative influence shapes cognitive health.

## The Dementia Conceptual Framework

**Figure 1:**
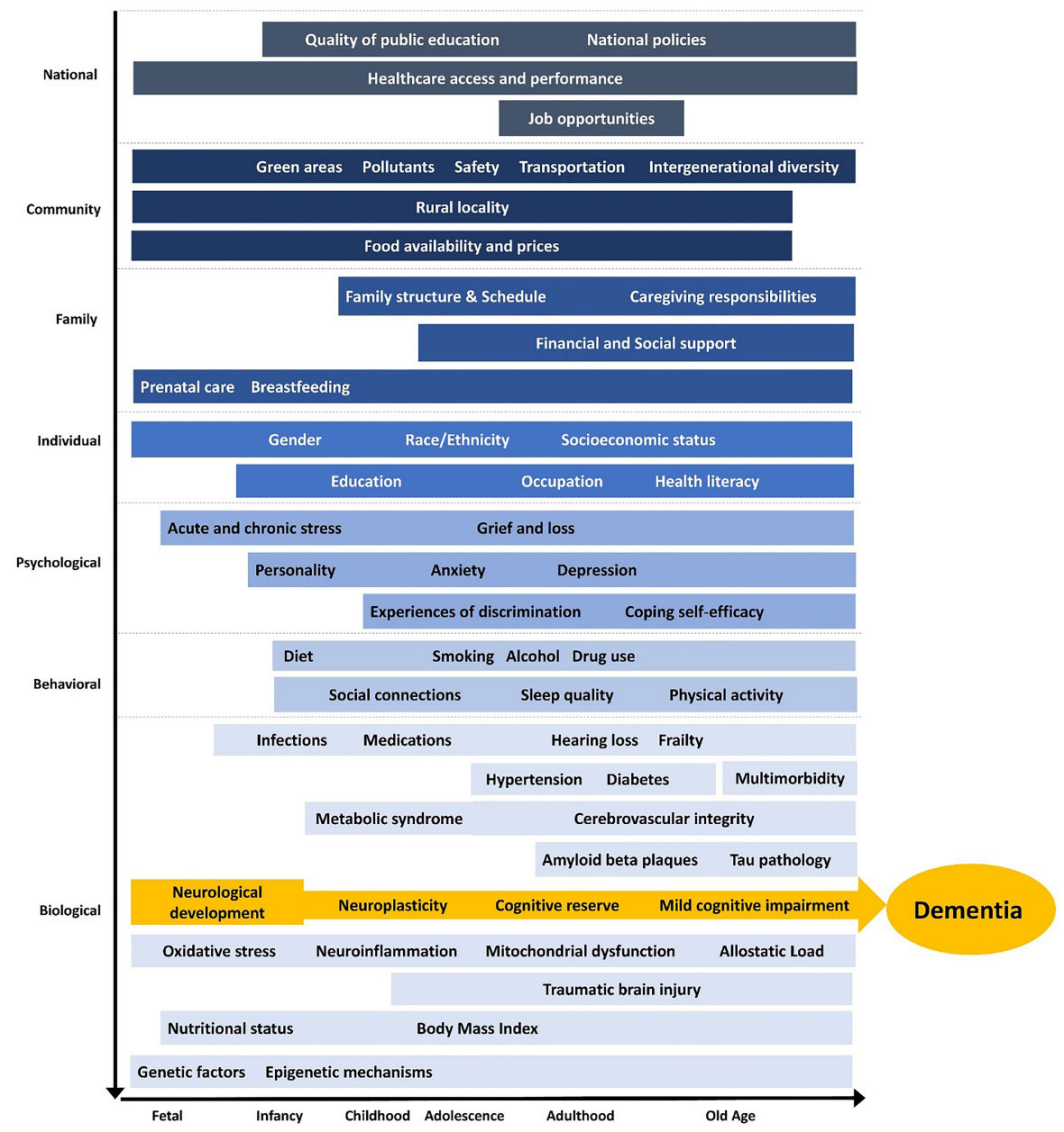
Multilevel conceptual framework for dementia.

The dementia conceptual framework presented here is underpinned by a bi-axial model that maps the relationship between the levels of influence and the stages of life in which they occur (Figure 1). The y-axis categorizes factors by levels - biological, behavioral, psychological, individual, family, community, and national - arranged hierarchically based on their proximity to and influence on the outcome. The x-axis represents the life course, beginning with fetal development on the left and progressing through infancy, childhood, adolescence, adulthood, and finally to old age, where the outcome dementia is located on the far right.

This model provides a structured view of how exposures at different life stages contribute to dementia. For example, early-life factors such as maternal nutrition and prenatal care influence neurological development, laying the foundation for neuroplasticity and cognitive reserve. In contrast, later-life factors such as sleep quality, frailty, and multimorbidity accelerate cognitive decline in old age. These stages are crucial for understanding the pathways that lead to dementia, as exposures and influences are cumulative, interacting over time to shape cognitive resilience or vulnerability (6).

To highlight the different levels of influence, the framework organizes the factors visually across life stages and levels of proximity to the outcome. The yellow-highlighted pathway from neurological development to dementia, linked to education, health literacy, and employment through cognitive reserve, is key in disease incidence and remains potentially modifiable through life-course interventions (8). The lighter shades in the diagram emphasize lower-level determinants, such as genetic factors and epigenetic mechanisms, which are more deeply rooted and harder to modify. In contrast, the darker colors represent more modifiable and broader factors, such as the quality of public education, community support systems, and health literacy, which can be targeted for interventions to delay or reduce dementia risk on a larger scale (9). This gradient helps differentiate between factors that are more fixed and individual and those that are more flexible and can be modified at the population level.

The framework provides a comprehensive understanding of how dementia develops over the lifespan by combining this visual representation with detailed textual explanations. The diagram illustrates complex relationships among factors at various life stages, while the text explains these connections in greater depth and suggests the most plausible pathways. This dual approach offers a more complete view of dementia, making it possible to identify critical intervention points across the life course and at different levels of influence (4).

## Multilevel factors contributing to dementia

### Biological

At the biological level, several factors beginning in early life and continuing through adulthood contribute to dementia risk. There is little evidence that early-life infections affect brain development, increasing the risk of cognitive impairment later in life (10). However, medications with ototoxic properties, which can damage the ear's hearing structures, may lead to hearing loss, a known contributor to cognitive decline and dementia risk in older age (11). As individuals age, genetic predispositions significantly impact dementia risk, with the APOE ε4 allele being strongly linked to Alzheimer's disease (12). Carriers of this gene variant exhibit faster amyloid deposition and neurodegeneration. Epigenetic modifications, influenced by environmental exposures, further modulate dementia risk by altering gene expression related to brain aging (13). Two key biological processes, mitochondrial dysfunction and oxidative stress, contribute to neurodegeneration. Mitochondrial damage impairs cellular energy production, while oxidative stress accelerates neuronal damage by increasing free radical production. Neuroinflammation, often referred to as inflammaging, exacerbates brain aging and promotes the development of neurodegenerative diseases (14). In addition to these factors, body mass index (BMI), particularly in midlife, is a critical determinant of dementia risk (15). Cerebrovascular integrity is another critical factor, as poor vascular health, driven by conditions like hypertension, diabetes, and atherosclerosis, reduces blood flow to the brain, leading to vascular cognitive impairment (16). The accumulation of amyloid beta plaques and tau tangles, hallmark features of Alzheimer's disease, further disrupts neuronal communication and accelerates cognitive decline (17). Traumatic brain injury, particularly repeated injuries, is a well-established risk factor for dementia, as it causes long-term damage to brain structures, increasing vulnerability to cognitive impairment (18). Finally, allostatic load, the cumulative stress burden on the body, worsens neuroinflammation and mitochondrial dysfunction, hastening brain aging and increasing dementia susceptibility (19). This mechanism is also associated with the accumulation of chronic diseases, known as multimorbidity, another risk factor that predisposes older adults to dementia.

### Behavioral

At the behavioral level, lifestyle choices made throughout life impact dementia risk (4). For example, dietary habits throughout life play a critical role in brain health. Diets rich in antioxidants and healthy fats, such as the Mediterranean diet, are associated with lower dementia risk, while poor dietary habits can increase oxidative stress and inflammation, contributing to cognitive decline (20). In adolescence and adulthood, behaviors such as smoking, alcohol consumption, and drug use can impair vascular health, increasing the likelihood of conditions like atherosclerosis, which affect cognitive function (21). Physical activity, on the other hand, has consistently been shown to protect against cognitive decline by enhancing neuroplasticity and promoting cerebrovascular health (9). Additionally, sleep quality plays a vital role in cognitive health. Poor sleep, particularly in adulthood and old age, has been linked to increased amyloid accumulation in the brain, accelerating the progression toward dementia (22). Social connections and maintaining strong social engagement throughout life are also critical behavioral factors. Social interaction stimulates cognitive functions and reduces isolation, which is linked to increased dementia risk (6).

### Psychological

Psychological factors, such as personality traits, anxiety, and depression, have been identified as significant contributors to dementia risk. Coping self-efficacy, or the belief in one's ability to manage life's challenges, also plays a role in resilience against cognitive decline (23). Stress, whether acute or prolonged, contributes to allostatic load, which exacerbates neuroinflammation and cognitive decline. This mechanism has been reported among those who experience different forms of discrimination (24). Grief and loss, often experienced in old age, also contribute to psychological burden and dementia.

### Individual

At the individual level, factors such as education, occupation, and health literacy are strongly linked to dementia risk. Educational attainment has been consistently associated with greater cognitive reserve, which delays the onset of dementia symptoms despite the presence of neuropathology (25). Gender, race/ethnicity, and socioeconomic status are also linked to dementia, with stronger effects in lower-income areas, with a higher burden on women and Black-identified people (26). Occupation and health literacy throughout life also play critical roles. Engaging in cognitively stimulating work and maintaining high levels of health literacy can protect against dementia by promoting brain activity and reducing the risk of diseases, such as hypertension and diabetes (27).

### Family

At the family level, family structure and caregiving responsibilities can influence dementia risk. This influence begins early in life, as prenatal nutrition and maternal health shape fetal brain development; when prenatal care is inadequate, fetal neurodevelopment may be compromised, increasing the likelihood of cognitive deficits later on (28). Breastfeeding during infancy has also been associated with better cognitive development, establishing the foundation for future cognitive reserve (29). Individuals with strong family support systems are more likely to maintain social engagement and mental well-being, which are protective factors against cognitive decline. However, caregiving responsibilities, particularly in later life, can increase stress levels, contributing to psychological and cognitive burdens (30).

### Community

At the community level, access to green spaces and community support systems promotes physical activity and social engagement, which protect against dementia. The availability of health resources and education systems that foster lifelong learning can also enhance cognitive reserve and reduce dementia risk across the population (31).

### National

At the national level, healthcare access and public education policies are pivotal in determining dementia outcomes (32). Countries with stronger healthcare systems and access to better preventive care are more effective at managing cardiovascular and metabolic risk factors, which are closely linked to cognitive decline (32,33). Public policies that promote education, health literacy, and social support systems contribute to healthier aging populations and reduce the incidence of dementia at the population level (33).

## Examples of pathways contributing to dementia

### Pathway 1: Cognitive reserve development across the life course

The first major pathway focuses on the development of cognitive reserve and its influence on dementia risk (8). Cognitive reserve refers to the brain's ability to adapt to damage by using existing cognitive networks or recruiting new ones (34). A higher cognitive reserve allows individuals to maintain cognitive function despite the presence of brain pathologies such as amyloid plaques or tau tangles (8). This pathway begins in the fetal stage at the family level, with maternal factors such as nutrition and prenatal care contributing to early brain development (35). As the child grows, factors at different levels, such as education, health literacy, social engagement, and physical activity during adolescence and adulthood, continue to build cognitive reserve. A key determinant is educational attainment, which has been shown to correlate strongly with cognitive reserve (25). It has been shown that older people with higher cognitive reserve are more likely to delay the onset of dementia symptoms, even in the presence of amyloid beta or tau pathology, which are important factors at the biological level (35). Thus, interventions aimed at increasing cognitive reserve, such as promoting lifelong learning, physical activity, and social engagement, are crucial for reducing the burden of dementia. Such interventions can be implemented at the community and national levels, impacting many people (36).

### Pathway 2: Cerebrovascular integrity and dementia risk

The second pathway emphasizes the role of cerebrovascular integrity in protecting against dementia (26). Cerebrovascular integrity refers to the health of the brain's blood vessels, which are critical for ensuring that the brain receives enough oxygen and nutrients (16). This biological level is shaped early in life by family-level exposures, such as prenatal health, which can influence blood vessel development. Throughout adolescence and adulthood, behavioral-level factors such as smoking and poor diet can disrupt vascular function at the biological level, raising the risk of hypertension, atherosclerosis, and stroke (16). These conditions compromise blood flow to the brain, leading to vascular cognitive impairment and the onset of dementia at advanced age (37). In older adults, maintaining cerebrovascular integrity is essential for preventing the acceleration of cognitive decline (38). Compromised vascular function exacerbates the impact of other pathologies at the biological level, such as amyloid plaques and tau tangles, on the brain, increasing the risk of dementia (39). Interventions at the behavioral level that focus on controlling cardiovascular risk factors, including blood pressure management, smoking cessation, and dietary improvements, can help preserve cerebrovascular integrity and reduce the risk of dementia (16). Policies at the national level, such as stricter regulations on cigarette and alcohol advertising, investment in green spaces for physical activity, and reducing taxes on natural foods compared to ultra-processed products, can also help reduce the risk of dementia (40).

## Conclusion

Dementia is a condition resulting from the interaction of multiple factors, including biological, behavioral, psychological, and social influences across the life course. While significant progress has been made through prospective observational studies and neuropathological data, the field continues to expand. It is important to note that numerous drug trials targeting various pathways have yielded negative results, with no clear cause-and-effect relationship established. In this context, the multilevel life-course perspective is even more critical, as it offers a comprehensive approach to understanding dementia.

## Data Availability

All data analyzed during this study are included in the references.

## References

[1] Livingston G, Huntley J, Liu KY, Costafreda SG, Selbaek G, Alladi S (2024). Dementia prevention, intervention, and care: 2024 report of the Lancet standing Commission. Lancet.

[2] Prince M, Wimo A, Guerchet M, Ali G, Wu Y, Prina M (2015). The global impact of dementia: an analysis of prevalence, incidence, cost and trends. World Alzheimer Report.

[3] Pierce AL, Kawas CH (2017). Dementia in the oldest old: beyond Alzheimer disease. PLoS Med.

[4] Chey J, Kwak S (2023). The life course approach to cognitive aging and dementia.

[5] Guerreiro R, Bras J (2015). The age factor in Alzheimer's disease. Genome Med.

[6] Whalley LJ, Dick FD, McNeill G (2006). A life-course approach to the aetiology of late-onset dementias. Lancet Neurol.

[7] Vernooij-Dassen M, Verspoor E, Samtani S, Sachdev PS, Ikram MA, Vernooij MW (2022). Recognition of social health: a conceptual framework in the context of dementia research. Front Psychiatry.

[8] Vance DE, Roberson AJ, McGuinness TM, Fazeli PL (2010). How neuroplasticity and cognitive reserve protect cognitive functioning. J Psychosoc Nurs Ment Health Services.

[9] Livingston G, Huntley J, Sommerlad A, Ames D, Ballard C, Banerjee S (2020). Dementia prevention, intervention, and care: 2020 report of the Lancet Commission. Lancet.

[10] Wiley CA (2021). Viral infection and dementia: a brief synthesis. Free Neuropathol.

[11] Cantuaria ML, Pedersen ER, Waldorff FB, Wermuth L, Pedersen KM, Poulsen AH (2024). Hearing loss, hearing aid use, and risk of dementia in older adults. JAMA Otolaryngol Head Neck Surg.

[12] Loy CT, Schofield PR, Turner AM, Kwok JB (2014). Genetics of dementia. Lancet.

[13] Sharma VK, Mehta V, Singh TG (2020). Alzheimer's disorder: epigenetic connection and associated risk factors. Curr Neuropharmacol.

[14] Wang X, Wang W, Li L, Perry G, Lee HG, Zhu X (2014). Oxidative stress and mitochondrial dysfunction in Alzheimer's disease. Biochim Biophys Acta.

[15] Li J, Joshi P, Ang TFA, Liu C, Auerbach S, Devine S (2021). Mid- to late-life body mass index and dementia risk: 38 years of follow-up of the Framingham study. Am J Epidemiol.

[16] Ferreira NV, Gonçalves NG, Szlejf C, Goulart AC, de Souza Santos I, Duncan BB (2024). Optimal cardiovascular health is associated with slower cognitive decline. Eur J Neurol.

[17] Nelson ME, Jester DJ, Petkus AJ, Andel R (2021). Cognitive reserve, Alzheimer's neuropathology, and risk of dementia: a systematic review and meta-analysis. Neuropsychol Rev.

[18] Shively S, Scher AI, Perl DP, Diaz-Arrastia R (2012). Dementia resulting from traumatic brain injury: what is the pathology?. Arch Neurol.

[19] Feng L, Ye Z, Du Z, Pan Y, Canida T, Ke H (2024). Association between allostatic load and accelerated white matter brain aging: findings from the UK Biobank. medRxiv [Preprint].

[20] Shannon OM, Ranson JM, Gregory S, Macpherson H, Milte C, Lentjes M (2023). Mediterranean diet adherence is associated with lower dementia risk, independent of genetic predisposition: findings from the UK Biobank prospective cohort study. BMC Med.

[21] Charakida M, Georgiopoulos G, Dangardt F, Chiesa ST, Hughes AD, Rapala A (2019). Early vascular damage from smoking and alcohol in teenage years: the ALSPAC study. Eur Heart J.

[22] Shi L, Chen SJ, Ma MY, Bao YP, Han Y, Wang YM (2018). Sleep disturbances increase the risk of dementia: A systematic review and meta-analysis. Sleep Med Rev.

[23] Tonga JB, Eilertsen DE, Solem IKL, Arnevik EA, Korsnes MS, Ulstein ID (2020). Effect of self-efficacy on quality of life in people with mild cognitive impairment and mild dementia: the mediating roles of depression and anxiety. Am J Alzheimer's Disr Demen.

[24] Bancks MP, Byrd GS, Caban-Holt A, Fitzpatrick AL, Forrester SN, Hayden KM (2023). Self-reported experiences of discrimination and incident dementia. Alzheimers Dement.

[25] Gonçalves NG, Avila JC, Bertola L, Obregón AM, Ferri CP, Wong R (2023). Education and cognitive function among older adults in Brazil and Mexico. Alzheimers Dement (Amst).

[26] Suemoto CK, Mukadam N, Brucki SMD, Caramelli P, Nitrini R, Laks J (2023). Risk factors for dementia in Brazil: differences by region and race. Alzheimers Dement.

[27] Oliveira D, Bosco A, di Lorito C (2019). Is poor health literacy a risk factor for dementia in older adults? Systematic literature review of prospective cohort studies. Maturitas.

[28] Cortés-Albornoz MC, García-Guáqueta DP, Velez-van-Meerbeke A, Talero-Gutiérrez C (2021). Maternal nutrition and neurodevelopment: a scoping review. Nutrients.

[29] Donovan M, Spatz DL (2024). Effects of a Person's lactation history on later-life development of Alzheimer's disease: an integrative review. Breastfeed Med.

[30] Brodaty H, Donkin M (2009). Family caregivers of people with dementia. Dialogues Clin Neurosci.

[31] Reuben A, Richmond-Rakerd LS, Milne B, Shah D, Pearson A, Hogan S (2024). Dementia, dementia's risk factors and premorbid brain structure are concentrated in disadvantaged areas: National register and birth-cohort geographic analyses. Alzheimers Dement.

[32] Hampel H, Vergallo A, Iwatsubo T, Cho M, Kurokawa K, Wang H (2022). Evaluation of major national dementia policies and health-care system preparedness for early medical action and implementation. Alzheimers Dement.

[33] WHO (World Health Organization) Global action plan on the public health response to dementia 2017-2025: World Health Organization; 2017. https://www.who.int/publications/i/item/global-action-plan-on-the-public-health-response-to-dementia-2017---2025.

[34] Pettigrew C, Soldan A (2019). Defining cognitive reserve and implications for cognitive aging. Curr Neurol Neurosci Rep.

[35] Liu Y, Lu G, Liu L, He Y, Gong W (2024). Cognitive reserve over the life course and risk of dementia: a systematic review and meta-analysis. Front Aging Neurosci.

[36] Crivelli L, Calandri IL, Suemoto CK, Salinas RM, Velilla LM, Yassuda MS (2023). Latin American initiative for lifestyle intervention to prevent cognitive decline (LatAm-FINGERS): study design and harmonization. Alzheimers Dement.

[37] Sierra C (2020). Hypertension and the risk of dementia. Front Cardiovasc Med.

[38] Kulshreshtha A, Goetz M, Alonso A, Shah AJ, Bremner JD, Goldberg J (2019). Association between cardiovascular health and cognitive performance: a twins study. J Alzheimers Dis.

[39] Leszek J, Mikhaylenko EV, Belousov DM, Koutsouraki E, Szczechowiak K, Kobusiak-Prokopowicz M (2021). The links between cardiovascular diseases and Alzheimer's disease. Curr Neuropharmacol.

[40] Hassen HY, Ndejjo R, Van Geertruyden JP, Musinguzi G, Abrams S, Bastiaens H (2022). Type and effectiveness of community-based interventions in improving knowledge related to cardiovascular diseases and risk factors: a systematic review. Am J Prev Cardiol.

